# Satisfaction, Usability, and Compliance With the Use of Smartwatches for Ecological Momentary Assessment of Knee Osteoarthritis Symptoms in Older Adults: Usability Study

**DOI:** 10.2196/24553

**Published:** 2021-07-14

**Authors:** Charlotte Rouzaud Laborde, Erta Cenko, Mamoun T Mardini, Subhash Nerella, Matin Kheirkhahan, Sanjay Ranka, Roger B Fillingim, Duane B Corbett, Eric Weber, Parisa Rashidi, Todd Manini

**Affiliations:** 1 Department of Pharmacy University of Toulouse Toulouse France; 2 Department of Aging and Geriatric research University of Florida Gainesville, FL United States; 3 Department of Epidemiology University of Florida Gainesville, FL United States; 4 Department of Biomedical Engineering University of Florida Gainesville, FL United States; 5 Google Mountain View California, CA United States; 6 Department of Computer and Information Science and Engineering University of Florida Gainesville, FL United States; 7 Department of Community Dentistry and Behavioral Science University of Florida Gainesville, FL United States

**Keywords:** ehealth, mobile health, ecological momentary assessment, real-time online assessment and mobility monitor, ROAMM, older adults, compliance, personal satisfaction, usability, smartwatch, knee osteoarthritis, pain, fatigue, wearable electronic device, mobile application

## Abstract

**Background:**

Smartwatches enable physicians to monitor symptoms in patients with knee osteoarthritis, their behavior, and their environment. Older adults experience fluctuations in their pain and related symptoms (mood, fatigue, and sleep quality) that smartwatches are ideally suited to capture remotely in a convenient manner.

**Objective:**

The aim of this study was to evaluate satisfaction, usability, and compliance using the real-time, online assessment and mobility monitoring (ROAMM) mobile app designed for smartwatches for individuals with knee osteoarthritis.

**Methods:**

Participants (N=28; mean age 73.2, SD 5.5 years; 70% female) with reported knee osteoarthritis were asked to wear a smartwatch with the ROAMM app installed. They were prompted to report their prior night’s sleep quality in the morning, followed by ecological momentary assessments (EMAs) of their pain, fatigue, mood, and activity in the morning, afternoon, and evening. Satisfaction, comfort, and usability were evaluated using a standardized questionnaire. Compliance with regard to answering EMAs was calculated after excluding time when the watch was not being worn for technical reasons (eg, while charging).

**Results:**

A majority of participants reported that the text displayed was large enough to read (22/26, 85%), and all participants found it easy to enter ratings using the smartwatch. Approximately half of the participants found the smartwatch to be comfortable (14/26, 54%) and would consider wearing it as their personal watch (11/24, 46%). Most participants were satisfied with its battery charging system (20/26, 77%). A majority of participants (19/26, 73%) expressed their willingness to use the ROAMM app for a 1-year research study. The overall EMA compliance rate was 83% (2505/3036 responses). The compliance rate was lower among those not regularly wearing a wristwatch (10/26, 88% vs 16/26, 71%) and among those who found the text too small to read (4/26, 86% vs 22/26, 60%).

**Conclusions:**

Older adults with knee osteoarthritis positively rated the ROAMM smartwatch app and were generally satisfied with the device. The high compliance rates coupled with the willingness to participate in a long-term study suggest that the ROAMM app is a viable approach to remotely collecting health symptoms and behaviors for both research and clinical endeavors.

## Introduction

Mobile devices are becoming commonplace in patient-based research [1]. Their ability to capture sensor data and enable interaction with participants in both observational and interventional studies makes mobile devices a powerful tool to augment traditional data collection approaches [2]. For example, these devices passively record activity with an accelerometer and location via GPS sensors to track physical activity and mobility. This information could be useful in understanding patients’ symptoms in the free-living environment. Such knowledge would be ideal for patients with osteoarthritis who exhibit variable pain experiences that may also interact with their mood and fatigue levels [3,4]. Coupled with sensor-based mobility data, smart devices offer a rich portrait of the interplay between symptoms and mobility levels.

Osteoarthritis is a degenerative and progressive disease affecting approximately 250 million patients worldwide [5]. Pain experiences greatly differ between patients and are often irregular within the same patient [6]. The complexity of symptoms is partly due to the site (knee, hip, or hand), genetic predisposition, initial cause of damage (ie, injury), obesity status, level of inflammation, and environmental factors [5,7]. Traditionally, patients receive treatment after reporting pain complaints and a physical examination along with optional imaging (eg, radiographs) [8,9]. Physical activity patterns, mobility function, and symptoms are used by clinical practitioners to inform treatment decisions [8,10,11]. However, difficulty in retrospective assessment of complex experiences like pain and the recall bias of self-assessing activity patterns present obstacles for care management of patients with osteoarthritis [12]. As a result, there has been considerable interest in using smart mobile devices—phones and wearables—for ascertaining symptoms and objective activity measures for informing practitioners [13]. In 2019, approximately 30 to 40 apps were designed for logging pain symptoms, but only one-fifth of those apps engaged the patients for which they were designed [14]. Moreover, none were solely designed for a smartwatch interface. Mobile devices and smart wearables have the potential to better characterize symptoms in the free-living world, but involvement of end-users (eg, patients) are necessary for appropriate design and long-term adoption.

New tools are needed to collect symptoms, experiences, and patterns of mobility and activity in real time in the free-living environment. Ecological momentary assessment (EMA) is a method based on data collection originally developed by Larson and Csikszentmihalyi in 1983 [15] for the psychological assessment of what activities people engage in, how they feel, and what they are thinking during their daily lives. It was developed because people are poor at reconstructing psychological experiences after they have occurred [16,17]. Rather, EMA considers experiences in the moment in a real-world environment and is potentially more representative of reality [18]. EMAs were first collected using paper diaries, followed by dedicated electronic diaries [19]. Recently, however, smartphone and smartwatch apps are becoming a pervasive means of assessing medical symptoms [20,21]. Work by Murphy and Smith demonstrated that tracking activity patterns with daily EMA fatigue reports yielded insights into the manifestation of activity-induced fatigue in participants with knee or hip osteoarthritis [22]. Another recent report used a custom-designed smartwatch app to prompt older adults with knee osteoarthritis to report their pain 4 to 5 times per day for approximately 3 months. Results demonstrated that older adults wore the watch for 75% of the study duration and answered 50% to 60% of the twice-daily prompts to rate their pain. Despite some drawbacks, including battery drain and technical issues, participants generally thought the watch was convenient and acceptable [23]. Although this previous work is encouraging, additional research is clearly needed to document smartwatch satisfaction, usability, and compliance for knee osteoarthritis symptoms.

The large increase in mobile medical apps has prompted the US Food and Drug Administration (FDA) to release a guidance statement [24]. The FDA is clearly supportive of evaluating patient-reported outcomes [25]; however, the framework for regulating medical mobile apps is still in its infancy [24]. Moreover, FDA guidance documents state that any patient-based software should undergo evaluation for overall design, usability, and acceptability for use in clinical care and research settings [26]. In that regard, the objective of our study was to evaluate satisfaction, usability, and compliance using the real-time and online assessment and mobility monitoring (ROAMM) mobile app designed for smartwatches. This study builds on initial input from interviews about the ROAMM app interface and usability in both patients and practitioners [27,28]. We hypothesized that older adults with knee osteoarthritis would provide positive satisfaction and usability ratings while being compliant with wearing the smartwatch and answering EMA prompts over an approximately 2-week evaluation period.

## Methods

### Participant Recruitment and Visit Design

Community-dwelling older adults aged 65 years and above with symptomatic unilateral or bilateral knee osteoarthritis were enrolled in the study. Recruitment sources included community advertisements and participant-based registries. Exclusion criteria included significant cognitive impairment, neurological conditions that severely inhibited mobility, inability to communicate because of severe hearing loss or speech disorder, terminal illness with life expectancy less than 12 months, severe pulmonary disease, renal failure with hemodialysis, severe psychiatric disorder (eg, bipolar, schizophrenia), excessive alcohol use (>14 drinks per week), drug addiction, or treatment for cancer (radiation or chemotherapy) within the past 1 year. All participants provided written informed consent, and the protocol was approved by the University of Florida Institutional Review Board.

Participants were asked to attend 2 clinic visits: one at baseline and another approximately 2 weeks later. After providing written informed consent, participants were administered the Mini-Mental Status Examination and then instructed on how to use the ROAMM app as previously described [27,29]. Participants were provided a simple user guide on how to use the wireless charging station and USB cable. They were also provided with a demographic questionnaire and an “exit” questionnaire that asked about their satisfaction with watch functionality and usability (see Multimedia Appendix 1) to be completed at the end of the second week. At the second visit, participants were asked to return the smartwatch and completed questionnaires.

### ROAMM App and EMA

The ROAMM app was developed at the University of Florida to enable real-time capture of patient-generated information. The smartwatch app collects wearable sensor (accelerometer and GPS) data simultaneously with symptom EMAs, as described previously [27]. Briefly, the ROAMM app is composed of a server and smartwatch app that are remotely connected through a secure https protocol. This integrated framework is designed and developed to perform several tasks, including remote data collection, storage, retrieval, and analysis. The primary goal of this project was to evaluate usability, satisfaction, and compliance of wearing the smartwatch and responding to EMA prompts in free-living conditions. Participants were asked to wear and charge the smartwatch every day for approximately 2 weeks during waking hours. The ROAMM app was programed to prompt the participant three times a day in a stratified random manner at prespecified windows: 8:00-11:59, 12:00-15:59, and 16:00-19:59.

**Figure 1:**
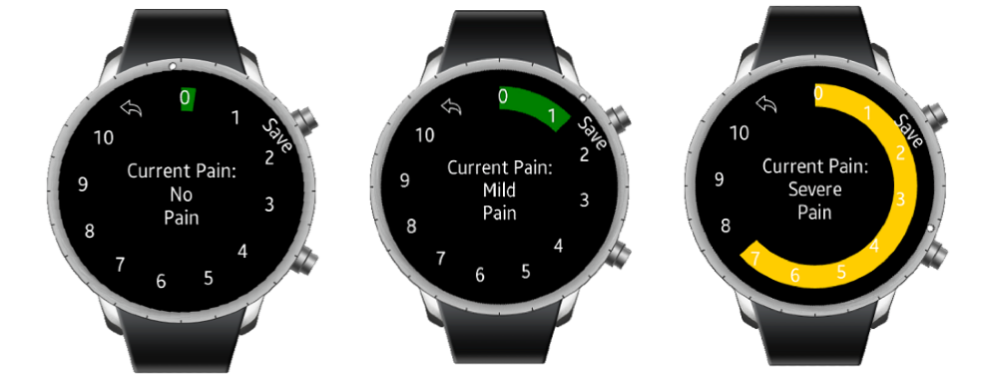
Depiction of watch face with visual analog scale used to rate pain intensity.

While wearing the watch, participants were prompted in the morning to report their prior night’s sleep quality. Thereafter, EMA pain, fatigue, mood, and activity were assessed throughout the day. Participants used the rotating bezel on the Samsung Gear S3 to dial in responses and then saved their responses by pressing a button located on top of the bezel. Rating scales were chosen based on the previous literature and the ability to scale down the content for the watch interface [30-34]. In the morning, participants rated their previous night’s sleep quality on a scale of 0 to 10 [35,36], with the following anchors: 0 to 1, “very poor”; 2, “poor”; 3 to 4, “OK”; 5 to 8, “well”; and 9 to 10, “very well”. EMA pain was evaluated using a valid and reliable numerical rating scale—the 11-point Box Scale (BS-11) of pain intensity that ranges from 0 to 10 [37,38]. There is a wide variety of versions of this scale and its inclusion of text anchors [39]. Because of the small watch face, we preferred to include more anchors than the traditional numeric scales. The following text anchors were shown as the participant rotated the dial: 0, “none”; 1 to 3, “mild”; 4 to 5, “moderate”; 6 to 7, “severe”; 8 to 9, “very severe”; and 10, “worst possible.” A depiction of the interface is shown in Figure 1 and in our previous publications [27,28].

Fatigue severity was also assessed using a scale of 0 to 10, using the abovementioned anchors, according to other similar validated scales previously reported [40-42]. Mood ratings were scaled slightly differently to more closely follow previously validated visual analogue scales [43,44]. By default, the zero value for “neutral” was placed at the bottom of the screen; rotation to the right reported negative mood ratings, with text anchors “negative” for –1 to –3 and “very negative” for –4 to –5. Rotation to the left reported positive mood ratings. Finally, participants rotated the bezel to choose an icon representing one of the following activity categories that they were presently engaged in: lying down, standing, walking, sitting, and other activities (representing other possible activities such as gardening and exercise). Thus, participants were prompted to report pain, fatigue, mood, and activity three times per day. To reduce burden, prompts were delivered in a contiguous manner—one after another. The total time to answer a set of prompts was very short, typically <30 seconds.

### ROAMM Exit Questionnaire to Evaluate Satisfaction and Usability

A 13-item exit questionnaire was administered at the end of the second week of the study (see questionnaire in Multimedia Appendix 1). The questions dealt with wearing comfort (eg, size, weight, wristband material), usability of the ROAMM app (eg, responding to prompts, font size, battery life), ease of using the inductive charger, and willingness to participate in future research studies. Participants were also asked to provide feedback to improve the app and its usability. Questions that used a 4-point Likert scale were reduced to two categories for statistical analysis (eg “very satisfied and satisfied” vs “somewhat satisfied and not satisfied”). Some questions asked participants to select as many options as possible that apply. Participants were also asked to provide any additional opinions of the ROAMM app and the smartwatch. Responses to this question were categorized into 4 major areas: technical issue; usability or functionality issue; size, weight, or display issue; and no issue (ie, positive opinion).

### ROAMM EMA Compliance

Compliance with each ROAMM app prompt was calculated in two ways. First, a raw compliance rate was calculated as the number of actual responses divided by the total number of possible responses assuming the watch was delivering the EMAs during programmed times:

(Total responses / Total number of possible responses) × 100.

Second, it was important to adjust the compliance rate to not *penalize* participants for potential technical issues or for when the watch was not being worn (ie, when charging). For this calculation, time windows with <3 hours of sensor data (ie, the watch was turned off during a time when an EMA could be delivered) or if the watch was charging for >30 minutes were flagged. Flagged time windows were not counted *against* the participant for nonresponsiveness (ie, they were not included in the denominator of the compliance rate). We considered this form of ”adjusted“ compliance in the stratified analysis described below. Only days where there were >3 hours of data, signifying a sufficient time to judge compliance, were considered in the analysis.

### Data Analysis

Comparisons of dichotomous responses on the patient satisfaction surveys were described as proportions and analyzed using Fisher exact test. Questions that contained multiple answers or free text were tallied, but formal statistical comparisons were not performed owing to the low number of responses. Adjusted compliances were compared using the Student *t* test between two groups and one-way analysis of variance with posthoc tests for more than two comparisons. Differences and associations were considered statistically significant at an α level <.05.

## Results

### Characteristics of the Study Population

Table 1 provides demographic characteristics of 27 of the 28 participants who completed the demographic questionnaire. Their mean age was 73.2 (SD 5.5) years, with a total of 19 (70%) female participants, 21 (78%) White participants, and 24 (89%) participants with a college-level education. Participants were moderately active, and most were overweight (n=10, 37%) or obese (n=9, 33%).

**Table 1:** Characteristics of study participants (N=27).

Characteristic				Participants
Age (years), mean (SD)				73.2 (5.5)
Sex, female, n (%)				19 (70)
**Race, n (%)**				
	White			21 (78)
	Other			6 (22)
**Education level, n (%)**				
	College education			24 (89)
	Other			3 (11)
**Living status, n (%)**				
	Lives alone			6 (22)
	Other			21 (78)
**Housing, n (%)**				
	Single-family home			22 (82)
	Other			5 (19)
**Morphology**				
	Height (m), mean (SD)			1.7 (0.1)
	Weight (kg), mean (SD)			80 (21.4)
	**BMI (kg/m^2^), mean (SD)**			28.3 (5.5)
		Obese (BMI ≥30 kg/m^2^), n (%)	9 (33)	
		Overweight (BMI 25-30 kg/m^2^), n (%)	10 (37)	
		Normal (BMI 18.5-25 kg/m^2^), n (%)	8 (30)	
**Physical activity, n (%)**				
	No regular leisure-time physical activity			4 (15)
	Some leisure-time physical activity			13 (48)
	Regular leisure-time physical activity			9 (33)
**Bill Payment, n (%)**				
	Somewhat difficult or very difficult time paying bills			13 (48)
	Not very difficult			14 (52)

### ROAMM Exit Questionnaire to Evaluate Satisfaction and Usability

Of the 26 participants, 81% (21) reported that they would be willing to wear the smartwatch while sleeping, and 85% (22) reported the text was large enough to read (Table 2). Moreover, all 26 participants reported it was easy to enter ratings using the smartwatch. About 77% (20/26) of the participants reported that the smartwatch’s battery life ended while they were wearing it. A similar proportion of participants regularly wore a wristwatch (16/26, 62% vs 10/26, 38%; *P*=.16) and answered that they would wear the smartwatch as their personal watch (11/24, 46% vs 13/24, 54%; *P*=.77).

Approximately half of the participants (14/26, 54%) reported the smartwatch was “very comfortable” or “comfortable” (Table 3). A follow-up question asking participants how the smartwatch comfort could be improved received the following responses: no changes (n=7), reduce weight of the watch (n=11), improve wristband clasp function (n=7), reduce display size (n=6), change the material of wrist band (n=6), reduce wrist band size (n=5), and other (size, weight, display and motion detection) (n=8). Despite these criticisms, a majority of the participants reported that they were satisfied with the function of the watch (19/26, 73%; *P*=.002) and charging the battery (20/26, 77; *P*<.001; Table 3).

**Table 2:** Real-time, online assessment and mobility monitoring exit questionnaire.

Question	Participants,^a^ n (%)		*P* value^b^
	Response: yes	Response: no	
Do you regularly wear a wristwatch?	16 (62)	10 (38)	.16
Would you wear the Samsung smartwatch as your personal watch? (n=24)	11 (46)	13 (54)	.77
For research purposes, would you occasionally wear the watch while sleeping?	21 (81)	5 (19)	<.001
Was the text large enough to read?	22 (85)	4 (16)	<.001
Was it easy to enter the ratings using the smartwatch?	26 (100)	0 (0)	N/A^c^
Did you charge it every night?	26 (100)	0 (0)	N/A
Did the watch ever run out of battery (ie, battery died) while you were wearing it?	20 (77)	6 (23)	<.001

^a^Total number of participants is 26, unless otherwise noted in the row header.

^b^Fisher exact test.

^c^N/A: not applicable.

**Table 3:** Real-time, online assessment and mobility monitoring exit questionnaire (continued).

Question		Participants (N=26), n (%)	*P* value^a^
**How satisfied were you with the function of the watch (ie, you were able to tell date/time easily)?**			.002
	Very satisfied and satisfied, n (%)	19 (73)	
	Somewhat satisfied and not satisfied, n (%)	7 (27)	
**How satisfied were you with the charging of the battery of the Samsung smartwatch?**			<.001
	Very satisfied and satisfied, n (%)	20 (78)	
	Somewhat satisfied and not satisfied, n (%)	6 (22)	
**How comfortable was the Samsung smartwatch to wear on a daily basis?**			.78
	Very comfortable and comfortable	14 (54)	
	Somewhat or not comfortable	12 (46)	
**How likely are you to participate in a 1-year research study asking you to wear the Samsung smartwatch daily?**			.002
	Very likely, likely or somewhat likely	19 (73)	
	Not likely	7 (27)	

^a^Fisher exact test.

Furthermore, a majority of the participants (19/26, 73%; *P*=.002) expressed their willingness to use the ROAMM app for a 1-year research study. In a follow-up question that asked the participants the reasons for responding ”not likely“ or ”somewhat likely“ (n=11), participants cited lack of comfort (n=5), (the watch was) not stylish (n=3), gets in the way (n=4), screen was hard to read (n=3), screen was unresponsive (n=4), privacy issue (n=1), technical issue (n=5), and size or weight issues (n=1). However, some of these participants were willing to wear the smartwatch for 1 month (n=5) or 3 months (n=1). Only 3 participants reported not willing to wear the watch at all.

All participants were asked to provide additional comments on the ROAMM app and the smartwatch. Those who opted to respond commented on technical issues (battery charging: n=10; temperature of the watch being too hot: n=2) and usability issues (resetting the watch: n=5; unresponsive screen: n=1; and size, weight, or display issues: n=7). There were positive opinions about the health monitoring aspects (n=4) and the ability to use the device as a phone or for email and calendar use (n=2).

### ROAMM EMA Compliance Rates

Twenty-eight participants wore the smartwatch for a mean of 13.9 (SD 0.4) days. When considering only those days with >3 hours of wear-time, participants wore the watch for a mean of 11.3 (SD 0.6) days. The accumulated total was 316 days recorded along with a total of 2505 smartwatch responses. The raw compliance rate was 61% (2505/4108) and the adjusted compliance rate was 83% (2505/3036). Specific to different windows throughout the day, the adjusted compliance rate was 86% (1004/1161) in the morning, 79% (800/1016) in the afternoon, and 77% (701/908) in the evening; details of adjusted compliance rate according to EMA responses in each window are shown in Table 4.

**Table 4:** Adjusted compliance rates according to ecological momentary assessment responses across the three evaluation windows.

Evaluation window	Sleep	Pain	Mood	Fatigue	Activity
Morning	.93	.875	.873	.87	.82
Afternoon	N/A^a^	.84	.823	.83	.71
Evening	N/A	.815	.795	.81	.71

^a^N/A: not applicable.

**Figure 2:**
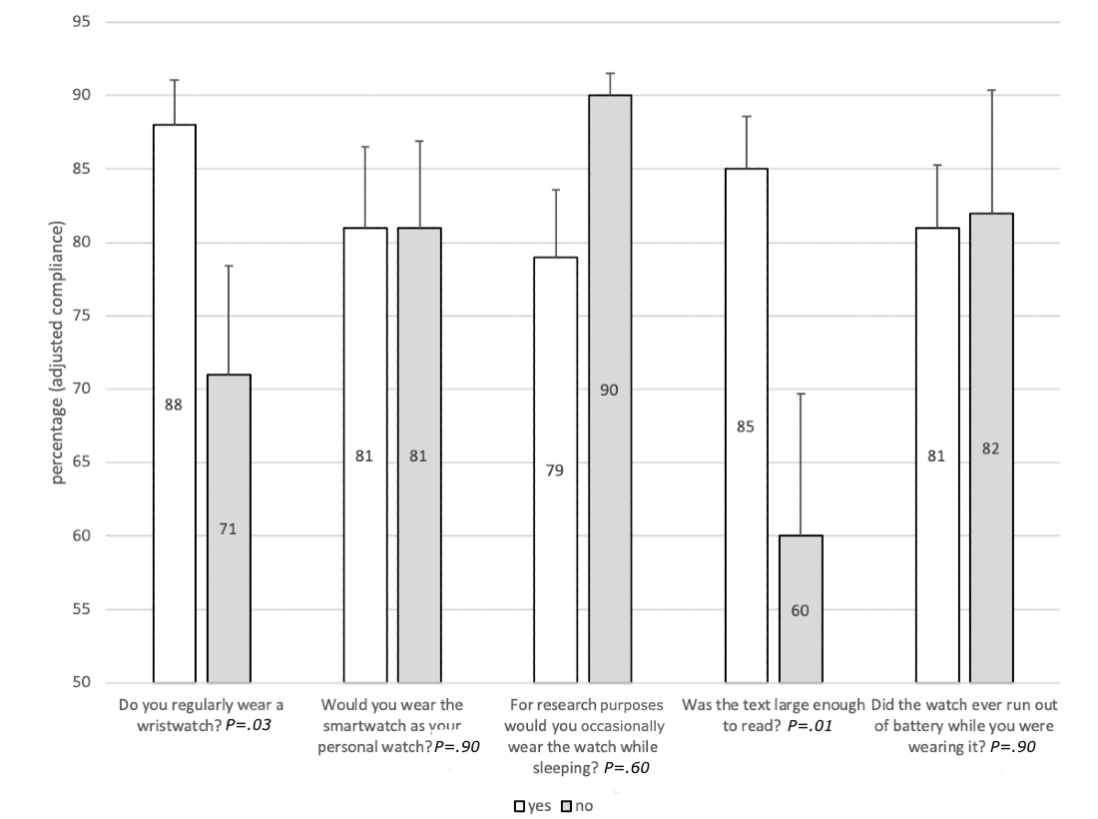
Adjusted compliance average according to responses from the real-time online assessment and mobility monitoring app exit questionnaire for Yes and No responses.

**Figure 3:**
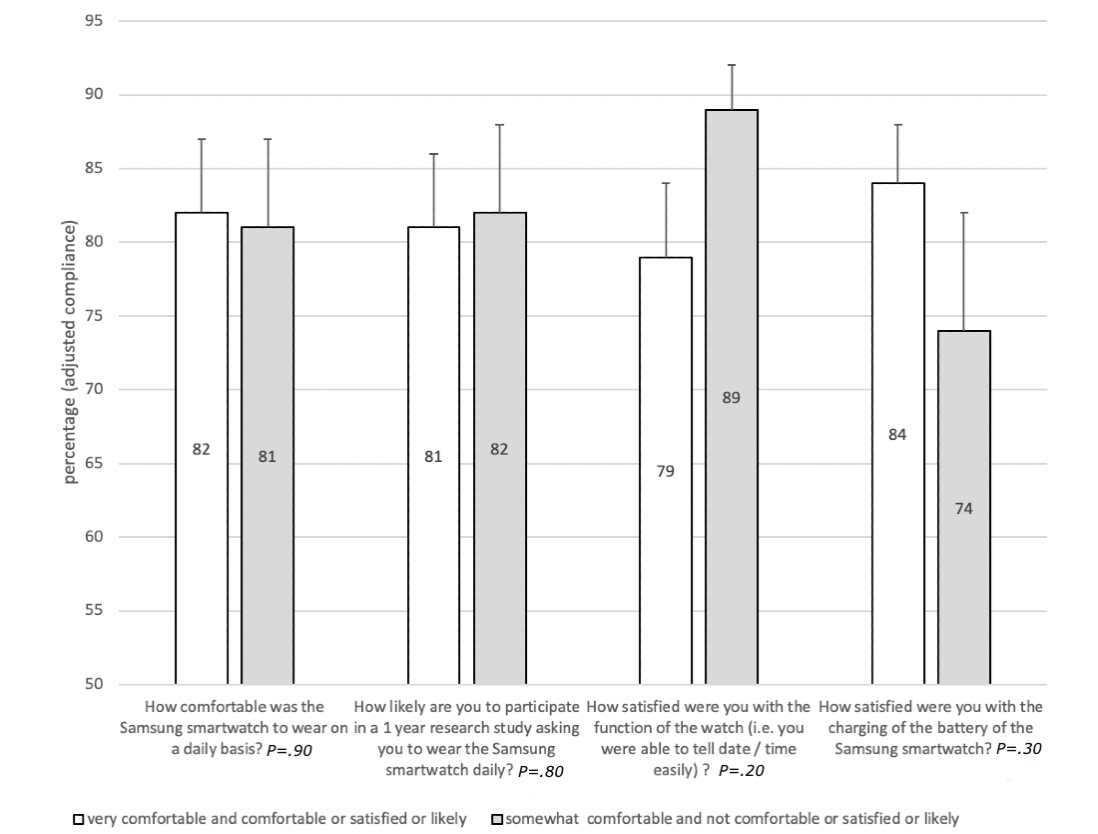
Adjusted compliance average according to responses from the real-time online assessment and mobility monitoring app exit questionnaire for Likert's responses.

Average adjusted compliance for EMA prompts were similar for pain, mood, fatigue, activity, and sleep (*P*=.14), although compliance was consistently lowest for reporting activity, which was the final question of the bundle. Moreover, average adjusted compliance rates were similar across the three time windows (*P*=.92). We explored potential reasons for compliance differences in a stratified analysis. Adjusted compliance was lower among those who do not regularly wear a wristwatch (88% vs 71%; *P*=.03) and was better among those who thought the text was large enough to read (86% vs 60%; *P*=.01) (Figure 2). No differences in adjusted compliance rates were observed for participants who reported higher satisfaction levels, those who were more likely to wear the watch for a 1-year study, those who would wear the smartwatch as a personal watch, and those who reported the smartwatch did run out of battery (Figures 2 and 3).

## Discussion

Gerontechnology is a relatively new concept that aims to promote health and well-being through technology that considers older adults’ needs and preferences [45]. The ROAMM app was developed based on these guiding principles and was designed to capture information about gerontological symptoms in the free-living environment. To ensure the technology is appropriate for this population, our research team and others have conducted focus groups to gather feedback about *gero-friendly* visualization (eg, display size) and functionality [27,46-49]. In the next phase of this study, we evaluated the technology in a small target sample. In this context, the purpose of this study was to evaluate the ROAMM smartwatch app for usability, satisfaction, and compliance in a patient population of older adults with knee osteoarthritis. Subsequent paragraphs interpret the results within the framework of gerontechnology and compare the current results to the existing literature. Based on our exit questionnaire, a majority of participants positively rated the ROAMM app display and functionality (eg, rotating dial). About half of the participants felt the smartwatch was uncomfortable, but almost three-fourths were likely to participate in a long-term study asking them to wear the smartwatch. Additionally, EMA compliance rates reported here were similar to a recent meta-analysis that pooled data from 701 participants across 12 EMA studies [50]. The high EMA compliance rates also indicate that older adults were able to use the app in free-living conditions. Participants also responded that it was easy to enter information using the rotating bezel, the text was sufficiently large, and they were satisfied with charging the smartwatch and effectively charging it every night. These responses culminated in a high likelihood of participating in research asking them to wear the smartwatch in a 1-year study—a goal for research related to health monitoring. However, it should be noted that willingness to participate in a long-duration study might not transfer to long-term compliance. Overall, our results suggest that older adults with knee osteoarthritis were generally satisfied with the ROAMM app and smartwatch, but the next intervention requires improved comfort and wearability for planning long-term studies.

Battery drain was a consistent issue observed during the study. The ROAMM app collects sensor data simultaneously with EMA data. We previously reported that the battery is most susceptible to the GPS sensor, with approximately 1% battery drain per collected sample [29]. This drain is exponentially increased when all sensors are collected simultaneously and further affected when the screen is activated during EMA responses. In a similar study, investigators from the KOALAP (Knee Osteoarthritis, Linking Activity and Pain) study also struggled to ensure the smartwatch battery lasted during the day—about 15 hours. They also found that the lack of battery life significantly impacted engagement with the smartwatch [51]. Additional innovation is needed on battery technology, smart sensor triggering (eg, activate accelerometer during movement only, activate GPS outside a geofence), and energy efficiency to ensure that apps like ROAMM are capable of health monitoring for an entire waking day. Advances in sensor technology and EMA tools for health monitoring are only effective if sufficient compliance is demonstrated [52]. The compliance rates reached in this study were consistent with systematic reviews of EMA for assessing chronic pain in adults (eg, 83% [53] and 86% [54]). However, achieving good compliance is a multifactorial challenge, as it involves the type of behavioral coaching, perceived burden, demographics of the population, and the usability of the technology [55]. Regarding the demographics, older adults tend to have higher compliance (88%-90% at 75 years old) than younger adults (72%-74% at 25 years old) even in technology-based evaluations, as reported in a chronic pain study [50]. In fact, an EMA-based study in older African American adults reported over a 90% compliance rate when rating their activity and stress, four times per day, on a smartphone [56]. There was also some evidence that fewer questions yielded higher compliance. We observed that a single sleep quality question in the morning yielded the highest compliance. In prior work, microinteraction EMAs—where people are prompted with fast, glanceable questions that could be answered in a few seconds similar to ROAMM—were developed on smartwatches and compared to less-frequent EMA prompts on smartphones. Researchers found that although prompts on the smartwatch were eight times more frequent than those on the smartphone, participants were 35% more compliant to short microinteraction EMAs on the smartwatch [57]. Participants also responded to EMAs in less time and reported the EMAs to be less distracting on the smartwatch than on the smartphone [58]. Therefore, EMAs on a smartwatch might serve as an excellent approach for longitudinal studies, which was also conveyed by a majority of older adults in our study who were willing to participate in a 1-year research study.

Stratified analysis of compliance rates yielded important information for practice and for planning future research. In general, compliance was similar between participants with different opinions of the comfort and satisfaction with the function of the smartwatch and ROAMM app. Unexpectedly, compliance was similar among participants not likely to wear the smartwatch as their own personal watch and those who would not volunteer for a 1-year research study. Participants regularly wearing a wristwatch had significantly higher compliance than nonwearers. Furthermore, individuals who had difficulty reading the text on the watch had lower compliance than those who did not experience difficulties. In the focus group study, approximately 80% of the respondents reported the display text size was adequate [27]. In the current study the same results were found (24/28, 79%) and participants reported the text was large enough. To be more inclusive and generalize to the population as a whole, future studies will need to consider whether people regularly wear watches and ensure text size or fonts are optimized for compliance.

There are strengths and weaknesses of this study that will aid in conducting future research using smartwatch devices for monitoring health. One of the weaknesses is that this study was performed on a relatively small, homogenous sample of older adults with knee osteoarthritis. In particular, this was a well-educated sample, and the results may not be generalizable to individuals with lower levels of education. Furthermore, we did not employ a commonly used ”usability“ scale for assessing the ROAMM app, which makes comparisons to the literature difficult. At the time of data collection, existing scales were not appropriate for assessing both the software and hardware of wearable devices. Moreover, despite internal pilot testing, rapid battery drainage found during wear in the free-living environment remained to be an issue. These weaknesses are balanced with some strengths such as the thorough investigation of usability and user compliance following an extended use of the ROAMM app in real-world settings.

In conclusion, older adults with knee osteoarthritis positively rated and were generally satisfied with the ROAMM app on the Samsung smart watch. Battery life remains a concern and will need to be carefully considered in future studies. Compliance rates were generally high but were impacted by personal experiences wearing a watch and text readability. After using the ROAMM app for about 2 weeks, a majority of older adults were willing to participate in a 1-year study requiring them to wear the smartwatch. Overall, the results support new opportunities to monitor health symptoms while capturing objective sensor information from a smartwatch in older adults with knee osteoarthritis.

## Appendix

Multimedia Appendix 1 Exit questionnaire to be filled in by the participants after 2 weeks.

## Abbreviations

DSAT: Data Science and Applied Technology
EMA: ecological momentary assessment
FDA: Food and Drug Administration
KOALAP: Knee Osteoarthritis, Linking Activity and Pain
ROAMM: real-time, online assessment and mobility monitoring
UF: University of Florida

## References

[1] Sama PR, Eapen ZJ, Weinfurt KP, Shah BR, Schulman KA (2014). An evaluation of mobile health application tools. JMIR Mhealth Uhealth.

[2] Peart DJ, Balsalobre-Fernández C, Shaw MP (2019). Use of mobile applications to collect data in sport, health, and exercise science: a narrative review. J Strength Cond Res.

[3] Allen KD, Coffman CJ, Golightly YM, Stechuchak KM, Keefe FJ (2009). Daily pain variations among patients with hand, hip, and knee osteoarthritis. Osteoarthritis Cartilage.

[4] Murphy SL, Kratz AL, Williams DA, Geisser ME (2012). The association between symptoms, pain coping strategies, and physical activity among people with symptomatic knee and hip osteoarthritis. Front Psychol.

[5] Lim SS, Vos T, Flaxman AD, Danaei G, Shibuya K, Adair-Rohani H, Amann M, Anderson HR, Andrews KG, Aryee M, Atkinson C, Bacchus LJ, Bahalim AN, Balakrishnan K, Balmes J, Barker-Collo S, Baxter A, Bell ML, Blore JD, Blyth F, Bonner C, Borges G, Bourne R, Boussinesq M, Brauer M, Brooks P, Bruce NG, Brunekreef B, Bryan-Hancock C, Bucello C, Buchbinder R, Bull F, Burnett RT, Byers TE, Calabria B, Carapetis J, Carnahan E, Chafe Z, Charlson F, Chen H, Chen JS, Cheng AT, Child JC, Cohen A, Colson KE, Cowie BC, Darby S, Darling S, Davis A, Degenhardt L, Dentener F, Des Jarlais DC, Devries K, Dherani M, Ding EL, Dorsey ER, Driscoll T, Edmond K, Ali SE, Engell RE, Erwin PJ, Fahimi S, Falder G, Farzadfar F, Ferrari A, Finucane MM, Flaxman S, Fowkes FGR, Freedman G, Freeman MK, Gakidou E, Ghosh S, Giovannucci E, Gmel G, Graham K, Grainger R, Grant B, Gunnell D, Gutierrez HR, Hall W, Hoek HW, Hogan A, Hosgood HD, Hoy D, Hu H, Hubbell BJ, Hutchings SJ, Ibeanusi SE, Jacklyn GL, Jasrasaria R, Jonas JB, Kan H, Kanis JA, Kassebaum N, Kawakami N, Khang Y, Khatibzadeh S, Khoo J, Kok C, Laden F, Lalloo R, Lan Q, Lathlean T, Leasher JL, Leigh J, Li Y, Lin JK, Lipshultz SE, London S, Lozano R, Lu Y, Mak J, Malekzadeh R, Mallinger L, Marcenes W, March L, Marks R, Martin R, McGale P, McGrath J, Mehta S, Mensah GA, Merriman TR, Micha R, Michaud C, Mishra V, Mohd Hanafiah K, Mokdad AA, Morawska L, Mozaffarian D, Murphy T, Naghavi M, Neal B, Nelson PK, Nolla JM, Norman R, Olives C, Omer SB, Orchard J, Osborne R, Ostro B, Page A, Pandey KD, Parry CDH, Passmore E, Patra J, Pearce N, Pelizzari PM, Petzold M, Phillips MR, Pope D, Pope CA, Powles J, Rao M, Razavi H, Rehfuess EA, Rehm JT, Ritz B, Rivara FP, Roberts T, Robinson C, Rodriguez-Portales JA, Romieu I, Room R, Rosenfeld LC, Roy A, Rushton L, Salomon JA, Sampson U, Sanchez-Riera L, Sanman E, Sapkota A, Seedat S, Shi P, Shield K, Shivakoti R, Singh GM, Sleet DA, Smith E, Smith KR, Stapelberg NJC, Steenland K, Stöckl Heidi, Stovner LJ, Straif K, Straney L, Thurston GD, Tran JH, Van Dingenen Rita, van Donkelaar Aaron, Veerman JL, Vijayakumar L, Weintraub R, Weissman MM, White RA, Whiteford H, Wiersma ST, Wilkinson JD, Williams HC, Williams W, Wilson N, Woolf AD, Yip P, Zielinski JM, Lopez AD, Murray CJL, Ezzati M, AlMazroa MA, Memish ZA (2012). A comparative risk assessment of burden of disease and injury attributable to 67 risk factors and risk factor clusters in 21 regions, 1990-2010: a systematic analysis for the Global Burden of Disease Study 2010. Lancet.

[6] Bartley EJ, Palit S, Staud R (2017). Predictors of osteoarthritis pain: the importance of resilience. Curr Rheumatol Rep.

[7] Hunter DJ, Guermazi A, Roemer F, Zhang Y, Neogi T (2013). Structural correlates of pain in joints with osteoarthritis. Osteoarthritis Cartilage.

[8] Kolasinski SL, Neogi T, Hochberg MC, Oatis C, Guyatt G, Block J, Callahan L, Copenhaver C, Dodge C, Felson D, Gellar K, Harvey WF, Hawker G, Herzig E, Kwoh CK, Nelson AE, Samuels J, Scanzello C, White D, Wise B, Altman RD, DiRenzo D, Fontanarosa J, Giradi G, Ishimori M, Misra D, Shah AA, Shmagel AK, Thoma LM, Turgunbaev M, Turner AS, Reston J (2020). 2019 American College of Rheumatology/Arthritis Foundation Guideline for the Management of Osteoarthritis of the Hand, Hip, and Knee. Arthritis Rheumatol.

[9] Hunter DJ, Bierma-Zeinstra S (2019). Osteoarthritis. Lancet.

[10] Juhl C, Lund H, Roos EM, Zhang W, Christensen R (2012). A hierarchy of patient-reported outcomes for meta-analysis of knee osteoarthritis trials: empirical evidence from a survey of high impact journals. Arthritis.

[11] Dobson F, Hinman RS, Hall M, Marshall CJ, Sayer T, Anderson C, Newcomb N, Stratford PW, Bennell KL (2017). Reliability and measurement error of the Osteoarthritis Research Society International (OARSI) recommended performance-based tests of physical function in people with hip and knee osteoarthritis. Osteoarthritis Cartilage.

[12] Daoust R, Sirois M, Lee JS, Perry JJ, Griffith LE, Worster A, Lang E, Paquet J, Chauny J, Émond M (2017). Painful memories: reliability of pain intensity recall at 3 months in senior patients. Pain Res Manag.

[13] Sim I (2019). Mobile devices and health. N Engl J Med.

[14] Zhao P, Yoo I, Lancey R, Varghese E (2019). Mobile applications for pain management: an app analysis for clinical usage. BMC Med Inform Decis Mak.

[15] Larson R, Csikszentmihalyi M (2014). The Experience Sampling Method. Flow and the Foundations of Positive Psychology.

[16] Bradburn NM, Rips LJ, Shevell SK (1987). Answering autobiographical questions: the impact of memory and inference on surveys. Science.

[17] Schwarz N (1999). Self-reports: How the questions shape the answers. Am Psychol.

[18] Smyth JM, Smyth JM (2003). Ecological momentary assessment research in behavioral medicine. J Happiness Stud.

[19] Shiffman S, Stone AA, Hufford MR (2008). Ecological momentary assessment. Annu Rev Clin Psychol.

[20] Boulos MNK, Brewer AC, Karimkhani C, Buller DB, Dellavalle RP (2014). Mobile medical and health apps: state of the art, concerns, regulatory control and certification. Online J Public Health Inform.

[21] McManus DD, Trinquart L, Benjamin EJ, Manders ES, Fusco K, Jung LS, Spartano NL, Kheterpal V, Nowak C, Sardana M, Murabito JM (2019). Design and preliminary findings from a new electronic cohort embedded in the Framingham heart study. J Med Internet Res.

[22] Murphy SL, Smith DM (2010). Ecological measurement of fatigue and fatigability in older adults with osteoarthritis. J Gerontol A Biol Sci Med Sci.

[23] Beukenhorst AL, Howells K, Cook L, McBeth J, O'Neill TW, Parkes MJ, Sanders C, Sergeant JC, Weihrich KS, Dixon WG (2020). Engagement and participant experiences with consumer smartwatches for health research: longitudinal, observational feasibility study. JMIR Mhealth Uhealth.

[24] (2019). Policy for Device Software Functions and Mobile Medical Applications. U.S. Food and Drug Administration (FDA).

[25] (2019). Patient-reported outcome measures: use in medical product development to support labeling claims. U.S. Food and Drug Administration (FDA).

[26] Quality System (QS) Regulation/Medical Device Good Manufacturing Practices Internet. U.S. Food and Drug Administration (FDA).

[27] Manini TM, Mendoza T, Battula M, Davoudi A, Kheirkhahan M, Young ME, Weber E, Fillingim RB, Rashidi P (2019). Perception of older adults toward smartwatch technology for assessing pain and related patient-reported outcomes: pilot study. JMIR Mhealth Uhealth.

[28] Alpert JM, Manini T, Roberts M, Kota NSP, Mendoza TV, Solberg LM, Rashidi P (2020). Secondary care provider attitudes towards patient generated health data from smartwatches. NPJ Digit Med.

[29] Kheirkhahan M, Nair S, Davoudi A, Rashidi P, Wanigatunga AA, Corbett DB, Mendoza T, Manini TM, Ranka S (2019). A smartwatch-based framework for real-time and online assessment and mobility monitoring. J Biomed Inform.

[30] Gould D, Kelly D, Goldstone L, Gammon J (2001). Examining the validity of pressure ulcer risk assessment scales: developing and using illustrated patient simulations to collect the data. Information point: visual analogue scale. J Clin Nurs.

[31] Paul-Dauphin A, Guillemin F, Virion JM, Briançon S (1999). Bias and precision in visual analogue scales: a randomized controlled trial. Am J Epidemiol.

[32] McCormack HM, Horne DJ, Sheather S (1988). Clinical applications of visual analogue scales: a critical review. Psychol Med.

[33] Huskisson EC (1974). Measurement of pain. Lancet.

[34] Downie WW, Leatham PA, Rhind VM, Wright V, Branco JA, Anderson JA (1978). Studies with pain rating scales. Ann Rheum Dis.

[35] Monk T, Reynolds C, Kupfer D, Buysse D, Coble P, Hayes A, Machen M, Petrie S, Ritenour A (1994). The Pittsburgh Sleep Diary. J Sleep Res.

[36] Smith MT, Wegener ST (2003). Measures of sleep: The Insomnia Severity Index, Medical Outcomes Study (MOS) Sleep Scale, Pittsburgh Sleep Diary (PSD), and Pittsburgh Sleep Quality Index (PSQI). Arthritis & Rheumatism.

[37] Jamison RN, Gracely RH, Raymond SA, Levine JG, Marino B, Herrmann TJ, Daly M, Fram D, Katz NP (2002). Comparative study of electronic vs. paper VAS ratings: a randomized, crossover trial using healthy volunteers. Pain.

[38] Farrar JT, Young JP, LaMoreaux L, Werth JL, Poole RM (2001). Clinical importance of changes in chronic pain intensity measured on an 11-point numerical pain rating scale. Pain.

[39] Hjermstad M, Fayers P, Haugen D, Caraceni A, Hanks G, Loge J, Fainsinger R, Aass N, Kaasa S, European Palliative Care Research Collaborative (EPCRC) (2011). Studies comparing Numerical Rating Scales, Verbal Rating Scales, and Visual Analogue Scales for assessment of pain intensity in adults: a systematic literature review. J Pain Symptom Manage.

[40] Hacker ED, Ferrans CE (2007). Ecological momentary assessment of fatigue in patients receiving intensive cancer therapy. J Pain Symptom Manage.

[41] Piper BF, Borneman T, Sun VC, Koczywas M, Uman G, Ferrell B, James RL (2008). Cancer-related fatigue: role of oncology nurses in translating National Comprehensive Cancer Network assessment guidelines into practice. Clin J Oncol Nurs.

[42] Curran SL, Beacham AO, Andrykowski MA (2004). Ecological momentary assessment of fatigue following breast cancer treatment. J Behav Med.

[43] Cella DF, Perry SW (1986). Reliability and concurrent validity of three visual-analogue mood scales. Psychol Rep.

[44] Folstein MF, Luria R (1973). Reliability, validity, and clinical application of the Visual Analogue Mood Scale. Psychol Med.

[45] Özsungur F (2020). Gerontechnological factors affecting successful aging of elderly. Aging Male.

[46] Mitzner TL, Savla J, Boot WR, Sharit J, Charness N, Czaja SJ, Rogers WA (2019). Technology adoption by older adults: findings from the PRISM trial. Gerontologist.

[47] Bergschöld JM, Neven L, Peine A (2020). DIY gerontechnology: circumventing mismatched technologies and bureaucratic procedure by creating care technologies of one's own. Sociol Health Illn.

[48] Chen K, Chan AHS (2014). Gerontechnology acceptance by elderly Hong Kong Chinese: a senior technology acceptance model (STAM). Ergonomics.

[49] Peek STM, Luijkx KG, Vrijhoef HJM, Nieboer ME, Aarts S, van der Voort CS, Rijnaard MD, Wouters EJM (2019). Understanding changes and stability in the long-term use of technologies by seniors who are aging in place: a dynamical framework. BMC Geriatr.

[50] Ono M, Schneider S, Junghaenel DU, Stone AA (2019). What affects the completion of ecological momentary assessments in chronic pain research? an individual patient data meta-analysis. J Med Internet Res.

[51] Beukenhorst AL, Parkes MJ, Cook L, Barnard R, van der Veer SN, Little MA, Howells K, Sanders C, Sergeant JC, O'Neill TW, McBeth J, Dixon WG (2019). Collecting symptoms and sensor data with consumer smartwatches (the Knee OsteoArthritis, Linking Activity and Pain Study): Protocol for a Longitudinal, Observational Feasibility Study. JMIR Res Protoc.

[52] NA (2017). Ecological momentary assessments and the science of behavior change. Exerc Sport Sci Rev.

[53] Morren M, van Dulmen Sandra, Ouwerkerk J, Bensing J (2009). Compliance with momentary pain measurement using electronic diaries: a systematic review. Eur J Pain.

[54] May M, Junghaenel DU, Ono M, Stone AA, Schneider S (2018). Ecological momentary assessment methodology in chronic pain research: a systematic review. J Pain.

[55] Hekler EB, Klasnja P, Traver V, Hendriks M (2013). Realizing effective behavioral management of health: the metamorphosis of behavioral science methods. IEEE Pulse.

[56] Fritz H, Tarraf W, Saleh DJ, Cutchin MP (2017). Using a smartphone-based ecological momentary assessment protocol with community dwelling older African Americans. J Gerontol B Psychol Sci Soc Sci.

[57] Intille S, Haynes C, Maniar D, Ponnada A, Manjourides J (2016). μEMA: microinteraction-based ecological momentary assessment (EMA) using a smartwatch. Proc ACM Int Conf Ubiquitous Comput.

[58] Ponnada A, Haynes C, Maniar D, Manjourides J, Intille S (2017). Microinteraction ecological momentary assessment response rates: effect of microinteractions or the smartwatch?. Proc ACM Interact Mob Wearable Ubiquitous Technol.

