# Supplementary material for: Satisfaction, Usability, and Compliance With the Use of Smartwatches for Ecological Momentary Assessment of Knee Osteoarthritis Symptoms in Older Adults: Usability Study
Source: JMIR Aging. 2021 Jul 14;4(3):e24553. doi: 10.2196/24553 (PMC8319786; doi:10.2196/24553)
Supplement: Multimedia Appendix 1 [file aging_v4i3e24553_app1.pdf]

EXIT QUESTIONNAIRE

ATTENTION!

Please complete this questionnaire before returning the study items, after wearing the smartwatch for two weeks, on the following date.

To Be Completed On:

--/--/----

UF | Institute on Aging
UNIVERSITY of FLORIDA

Your opinions about the Samsung smartwatch matter

Please answer the following questions about wearing the Samsung smartwatch. Please answer honestly because your answers will help us plan for the future of wearable technology dedicated to the health of older adults.

1. Do you regularly wear a wristwatch?

☐ Yes ☐ No

2. Would you wear the Samsung smartwatch as your personal watch without being asked to wear it as part of a research study?

☐ Yes ☐ No

3. How comfortable was the Samsung smartwatch to wear on a daily basis?

☐ Very comfortable ☐ Comfortable

☐ Somewhat comfortable ☐ Not comfortable

4. What would you change to improve the comfort of wearing the Samsung smartwatch?

☐ No changes needed

☐ Reduce wristband size

☐ Improve wristband clasp function

☐ Reduce weight of the watch

☐ Reduce display size

☐ Change the material of the wristband (cloth, metal etc.)

☐ Other ______________________

5. How likely are you to participate in a one-year research study asking you to wear the Samsung smartwatch daily?

☐ Very likely ☐ Likely

☐ Somewhat likely ☐ Not likely

5a. If your answer was “somewhat likely” or “not likely”, what is the most amount of time you would agree to wear the Samsung smartwatch?

☐ 1 month ☐ 3 months

☐ 6 months ☐ 9 months ☐ None

5b. If you answered “somewhat likely” or “not likely” to participate, what are some of the reasons for you answer (you can check more than one choice)?

☐ Uncomfortable (e.g. too tight or too loose)

☐ Not stylish

☐ Too bulky

☐ Too fragile

☐ It gets in the way

☐ The screen is hard to read

☐ The screen was not responsive

☐ Other (please write below any other reasons)

____

____

6. For research purposes, would you occasionally wear the watch while sleeping? How likely would you be to participate in a study that asked you to occasionally wear the watch while sleeping?

☐ Yes ☐ No

7a. Was the text large enough to read?

☐ Yes ☐ No

8.Was it easy to enter the ratings using the smartwatch?

☐ Yes ☐ No

8.a If ‘No’, can you provide more details?

9. Did you charge it every night?

☐ Yes ☐ No

10. Did the watch ever run out of battery (i.e. battery died) while you were wearing it?

☐ Yes ☐ No

10.a If ‘Yes’, how many times? _____ times

11. How satisfied were you with the function of the watch (i.e. you were able to tell date/time easily)?

☐ Very satisfied ☐ Satisfied

☐ Somewhat satisfied ☐ Not satisfied

12. How satisfied were you with charging the battery of the Samsung smartwatch?

☐ Very satisfied ☐ Satisfied

☐ Somewhat satisfied ☐ Not satisfied

13. Are there any other opinions about the Samsung smartwatch that you would like to share with us?

____

____

____
